# PP72 Technical Reports On Judicialized Health Technologies For Rare Diseases: Cross-Sectional Study

**DOI:** 10.1017/S0266462325102900

**Published:** 2025-12-29

**Authors:** Ana Luiza Cabrera Martimbianco, Verônica Colpani, Isabela Porto De Toledo, Camila Monteiro Cruz, Carolina de Oliveira Cruz Latorraca, Cecília Menezes Farinasso, Patrícia do Carmo Silva Parreira, Rafael Leite Pacheco, Roberta Borges Silva, Rachel Riera

## Abstract

**Introduction:**

Health judicialization is an escalating issue, especially in rare diseases where high treatment costs and complex management often limit treatment access. In Brazil, the National Council of Justice created support centers to provide technical scientific reports (TRs) based on health technology assessment (HTA) for evaluating judicialized health technologies. This study aimed to describe the frequency and characteristics of TRs related to rare diseases within this framework.

**Methods:**

This descriptive cross-sectional study was developed at the Center of HTA, Hospital Sírio-Libanês, Sao Paulo, Brazil. Data were collected from the National System of Technical Reports, which compiles evidence-based scientific information to support judicial decision-making. We included TRs submitted to the platform between 2019 and 2024. Extracted data were categorized based on the most frequent rare diseases estimated by the Orphanet database list of 2024, identified according to the International Classification of Diseases, 11th Revision coding manual. Additionally, the requested health technologies were identified, and quantitative analyses were performed to identify distribution patterns.

**Results:**

A total of 235,546 TRs were analyzed. The most prevalent judicialized rare diseases and their corresponding technologies were as follows: motor neuron disease (n=501), with 42 percent of requests involving riluzole or tauroursodeoxycholic acid; cystic fibrosis (n=415), with 60 percent of requests for elexacaftor, tezacaftor, and ivacaftor; muscular dystrophy (n=349), with 30 percent of requests for ataluren; Gaucher disease (n=183), with 42 percent of requests for agalsidase alfa; and amyloidosis (n=93), with 58 percent of requests for tafamidis meglumine or daratumumab.

**Conclusions:**

This analysis of TRs related to rare diseases highlighted the need for targeted interventions to improve access for the most prevalent conditions. By identifying the most frequently requested health technologies, this study emphasized the importance of evidence-based decision-making in addressing the challenges posed by rare diseases within the judicial system, particularly the high costs associated with their treatment.

